# Predicting Activity Duration in Smart Sensing Environments Using Synthetic Data and Partial Least Squares Regression: The Case of Dementia Patients

**DOI:** 10.3390/s22145410

**Published:** 2022-07-20

**Authors:** Miguel Ortiz-Barrios, Eric Järpe, Matías García-Constantino, Ian Cleland, Chris Nugent, Sebastián Arias-Fonseca, Natalia Jaramillo-Rueda

**Affiliations:** 1 Department of Productivity and Innovation, Universidad de la Costa CUC, Barranquilla 08002, Colombia; sarias9@cuc.edu.co (S.A.-F.); njaramil@cuc.edu.co (N.J.-R.); 2 Department of Intelligent Systems and Digital Design, Halmstad University, P.O. Box 823, S 301 18 Halmstad, Sweden; eric.jarpe@hh.se; 3 School of Computing, Computer Science Research Institute, Ulster University, Belfast BT37 0QB, UK; m.garcia-constantino@ulster.ac.uk (M.G.-C.); i.cleland@ulster.ac.uk (I.C.); cd.nugent@ulster.ac.uk (C.N.)

**Keywords:** activities of daily living (ADLs), activity recognition, activity duration, partial least square regression (PLSR), people with dementia (PwD), simulated data, artificial intelligence, sensor systems, smart homes

## Abstract

The accurate recognition of activities is fundamental for following up on the health progress of people with dementia (PwD), thereby supporting subsequent diagnosis and treatments. When monitoring the activities of daily living (ADLs), it is feasible to detect behaviour patterns, parse out the disease evolution, and consequently provide effective and timely assistance. However, this task is affected by uncertainties derived from the differences in smart home configurations and the way in which each person undertakes the ADLs. One adjacent pathway is to train a supervised classification algorithm using large-sized datasets; nonetheless, obtaining real-world data is costly and characterized by a challenging recruiting research process. The resulting activity data is then small and may not capture each person’s intrinsic properties. Simulation approaches have risen as an alternative efficient choice, but synthetic data can be significantly dissimilar compared to real data. Hence, this paper proposes the application of Partial Least Squares Regression (PLSR) to approximate the real activity duration of various ADLs based on synthetic observations. First, the real activity duration of each ADL is initially contrasted with the one derived from an intelligent environment simulator. Following this, different PLSR models were evaluated for estimating real activity duration based on synthetic variables. A case study including eight ADLs was considered to validate the proposed approach. The results revealed that simulated and real observations are significantly different in some ADLs (*p*-value < 0.05), nevertheless synthetic variables can be further modified to predict the real activity duration with high accuracy ($R^{2}\left(pred\right)>90\%$).

## 1. Introduction

Recent advances in medicine and healthcare have contributed to the increase in life expectancy worldwide, which has also brought more incidence of neurodegenerative conditions, such as dementia, in the elderly population. As reported by the World Health Organisation (WHO) in 2022, there are more than 55 million people living with dementia around the world, with an increase of nearly 10 million cases per year [1]. While the COVID-19 pandemic brought wider public attention to the challenges that health systems and carers (formal and informal) of people with dementia (PwD) face, these challenges were already acknowledged before the pandemic [2]. Technological means in the form of mobile apps and sensors to monitor the activities of daily living (ADLs) of PwD detect their behaviour patterns and emergency situations that require effective and timely human interventions have been more widely used to support their carers with good results [3].

Sensor-based ADLs monitoring solutions typically perform well in controlled laboratory conditions; however, in many cases, these solutions do not perform as expected in real-world conditions, such as in care homes [4], mainly because of uncertainties derived from smart home configurations and the different ways in which people perform ADLs. In this sense, while collecting real-world data might allow the development of more personalised approaches for each individual, this data collection is usually costly and characterized by a challenging recruiting research process, in which PwD may not be willing to adopt technological solutions [5]. In many sensor data collection instances that are part of research projects, there is usually not sufficient data to be analysed in such a way that has personalised benefits for the PwD involved. The use of synthetic data generated through simulation has been more frequently used as an alternative efficient choice to complement real datasets [6], overcoming the lack of sufficient real-world data and providing the capability of producing as much synthetic data as required for machine learning algorithms to generate personalised solutions. However, synthetic data can be significantly dissimilar compared to real data in the sense that while the generated data may be proportional and within acceptable ranges in general, they might not be an accurate and realistic representation of the specific cases of the PwD aimed at. Hence, the synthetic data would be an inaccurate alternative to data otherwise collected for each PwD and could lead to misleading and erroneous conclusions about the types, frequency, and duration of ADLs performed by PwD.

This paper proposes the application of Partial Least Squares Regression (PLSR) to approximate the real activity duration of various ADLs based on synthetic observations. The approach presented involves two phases: (i) comparing the duration of the ADLs that were collected from PwD (real data) with data derived from an intelligent environment simulator (synthetic data), and (ii) evaluating different PSLR models to estimate real activity duration of synthetic data. The criteria considered to estimate the real activity duration are: (i) number of events per activity (NEPA), (ii) number of events per sensor per activity (NEPSA), and (iii) activity duration. A case study is presented to evaluate this and it considers eight ADLs, which are deemed to be common activities performed by most people: (i) stay in bed, (ii) use restroom, (iii) make breakfast, (iv) get out of home, (v) get cold drink, (vi) stay in the office, (vii) get hot drink, and (viii) cook dinner. Note that while for healthy people without disabilities performing the ADLs considered may be easier to perform than for PwD, a machine learning personalised approach would be of benefit for both types of people in the prediction of activity duration, which in many cases can be related to health problems that could worsen if unattended, such as neurodegenerative diseases or infections. For example, inaccurate synthetic data about the frequency and duration of the “use of bathroom” ADL could present a higher frequency and a large duration that could then result in the carer or nurse of the PwD increasing the use of a drug for Urinary Tract Infection (UTI).

The main research question of our investigation is if statistical methods, such as PLSR, can be used on synthetic data to reliably approximate and predict the duration of ADLs performed by PwD. Therefore, the novelty of our approach is that it can use synthetically generated data to provide accurate personalised predictions on ADLs performed by PwD, significantly reducing the time in which a prediction model would be generated using just real-world data. This is because in the case of real-world data, it typically takes longer and involves more costs (staff salaries, participants’ recruitment, and use of equipment) to perform the collection, pre-processing, and formatting of the data so it can be used by statistical methods to then obtain predictions.

The research contributions of this paper are: (i) producing predictive models that provide a more accurate transformation of simulated data to describe duration of real activities, and (ii) identifying the main synthetic predictors for real activity duration in each ADL and its intrinsic properties, and how this metric varies amongst users. This study aims to bridge gaps of previous research works in contrasting real and synthetic data, for instance, the SynSys algorithm proposed by [7] does not take into account the nature of each ADL, which our approach does. It is intended that the model will be improved over time as more real-world data is collected for each PwD while providing a solution based on synthetic data that can provide early benefits to PwD. In practice, our approach would also reduce the costs involved in using ambient and wearable sensors for extended periods of time to collect real-world data from PwD, as well as costs involved with sensor maintenance, and data collection, pre-processing, and analysis.

The remainder of this paper is organised as follows: Section 2 presents the related works on ADLs and the generation of synthetic data. Section 3 presents the materials and methods used. The experiment definition is described in Section 4. Section 5 presents the results obtained and the discussion. Finally, Section 6 presents the conclusions.

## 2. Related Works

The ability to accurately recognise activities is central to many intelligent systems including smart home automation, ambient assisted living, assistive robotics and human computer interaction. For people living with dementia, activity recognition (AR) techniques have facilitated several use cases including detection of agitation [8], tracking cognitive impairment [9], detecting anomalous activities [10], detecting urinary tract infections [11], and mapping social interactions. This section provides a brief overview of the challenges associated with activity recognition within smart environments before discussing the potential of synthesised data to address this.

### 2.1. Synthesizing Data for Sensor Based Activity Recognition

AR research can be categorised into two main approaches; namely video or sensor-based. Sensor-based AR lags similar fields largely due to a lack of large-scale, high-quality, multi-modal, and labelled datasets. This has impeded progress in developing robust and generalised Machine Learning (ML) approaches. The success of supervised ML relies primarily on the availability of large datasets with high-quality annotations. Collecting labelled data or employing experts to label large datasets are infeasible in resource-constrained settings, such as healthcare. Indeed, the collection of human activity and behaviour data is time consuming, costly, and often limited in terms of availability.

To further complicate this issue, owing to biological and environmental factors, the same activity can be performed differently by different individuals. These differences are further enhanced when considering the variation that occurs in those with movement disorders (cerebral palsy, post-stroke) or cognitive impairment (dementia). Despite the amount of research undertaken in the field, issues such as cross-subject variability are still posing an obstacle to the deployment of solutions in large-scale and free-living settings.

To address these problems, researchers have been investigating the use of synthetic data for training, testing, and validation of ML-based AR techniques [7]. Early research in this area typically relied upon mathematical models such as Markov chains or Petri networks [12]. This then progressed to investigate combinations of approaches to model more complex activities. For example, Helal et al. [13] developed a solution using Markov chains to model activity patterns and combined this technique with Poisson distribution to generate realistic timestamps. This work later progressed to a software solution to allow researchers to create synthetic datasets.

More recently, researchers have been investigating the use of Generative Adversarial Networks (GANs) to generate synthetic sensor data. This approach utilises an adversarial discriminative model to determine whether a generated synthetic sample follows the same distribution as real data. The generative model continuously improves the quality of the synthetic data until the adversary is unable to distinguish real from synthetic data. Whenever large amounts of labelled data are unavailable, synthetic data can be generated to augment the available labelled data to provide enough data for training and testing. Using GANs, Alharbi et al. [14] explored and constructed a model for generating several types of human activity sensor data. They assessed the use of synthetic data to train two commonly-used classifier models, Convolutional Neural Network (CNN) and Long Short Term Memory (LSTM). In doing so, they demonstrated the efficacy of the proposed method on two publicly available human activity datasets, the Sussex-Huawei Locomotion (SHL) and Smoking Activity Dataset (SAD). The solution achieved improvements for both SHL (0.85 to 0.95 F1-score), and for SAD (0.70 to 0.77 F1-score) when using a CNN activity classifier.

### 2.2. Challenges with Existing Approaches for Data Synthesis

As summarised above, existing approaches for synthesising realistic data have shown great promise in improving the accuracy of ML approaches for AR. An ongoing challenge, however, in developing such solutions is the ability to generate simulated data that accurately represents real data. A comparison between real data collected within the Gator Tech Smart House and simulated data generated by Persim 3D [15] revealed average data similarities of between 78% and 81%. Another study comparing real data with data generated using the simulator MASSHA found the similarity to be between 88.10% and 93.52% in terms of frequency, and 98.27% and 99.09% in terms of duration on datasets containing single user activities [16].

Smart home data has a number of unique characteristics in comparison to most sequence and time-series data. Sensor data for activity recognition are typically not independently and identically distributed and exhibit both high dimensionality and a high level of complexity. Therefore, synthetic data must be generated that are consistent with these characteristics. Firstly, within a smart home, data do not typically arrive at a constant rate. Therefore, synthetic data must include realistic time stamps for each sensor reading. Secondly, many smart home sensors do not produce continuous values. Therefore, when synthesising data, a sensor name must be generated along with a corresponding value. For light or temperature sensors, the corresponding value is numeric at a set sample rate. For motion or door sensors, the corresponding value is binary (e.g., ON or OFF). Finally, sensor readings are accompanied by corresponding activity labels. In real experiments, activity labels are provided by external annotators. The label must therefore also be generated and attributed to the correct set of sensors, activating in the correct sequence and over the right time frame.

To address these complexities, Dahmen and Cook [7] developed SynSys. This solution semi-supervised learning combined with synthetic data generation to generate synthetic data could be used to improve the accuracy of ML approaches for AR. They showed that the SynSys algorithm was able to successfully generate more realistic synthetic data for a week of smart home data in comparison to random data, data from another home, and synthesised data created using a single Hidden Markov Model (HMM) and Poisson regression technique. Combining real data with synthetic data generated from SynSys was shown to significantly improve the accuracy of activity recognition. The authors also noted that as SynSys is based on generating nested sequences of data and sequences of timestamps that capture duration, the method can be applied to more generalized data beyond the smart home datasets we used in our experiments.

Based on the existing literature, it has been noted that research comparing real observations and simulated data is largely limited and poorly developed. Alshammari et al. [17] reviewed 228 software tools for simulation and found that only a small number focused on generating datasets with the majority being focused on visualisation and context-awareness applications. Similarly, Bouchabou et al. [18] highlighted that whilst simulation tools provide an excellent opportunity to quickly generate and visualise smart home date; in particular, it is noted that synthetic datasets allow for quick evaluation of different sensor configurations within the environment without the requirement of physical deployment and volunteer subjects. Additionally, the annotation can be more precise compared to real dataset methods. Nonetheless, synthetic data can be un-realistic in comparison to real-world datasets. Typically, activities provided within synthetic datasets are less realistic in terms of execution rhythm and variability. Furthermore, the design of the virtual smart homes can be cumbersome for a non-expert designer. Bouchabou et al. [18] also noted that there are currently no publicly available synthetic datasets, though a number of open simulators, such as OpenSH, are available. In spite of the strong demand from ML surprisingly little has been done about assessing the validity of simulation-based studies and methodology for real data problems in image analysis and computer vision [19] and in a more general perspective [20]. In fact, Kleijnen [21] argues that measurements for verification and validation of simulation models are in need of development.

As reported in this section, several approaches (Markov chains, software solutions like the PerSim3D and the MASSHA activity simulators, GANs, and SynSys) have addressed the generation of synthetic data with different levels of success in relation to how close the synthetic data was with real data as measured using different metrics. While the authors of the related works presented in this section highlight positive results, it is necessary to have a more standardised way (benchmark datasets, use same metrics, etc.) to determine their effectiveness and their limitations. This paper bridges this gap by (i) evaluating the equivalence between real and synthetic activity duration, and (ii) implementing PLSR to better approximate real activity duration based on simulated observations.

## 3. Materials and Methods

The data used in this study consists of actual observations of duration times for different daily activities and synthetic data mimicking the probabilistic properties of the real-world data. Please, refer to Section 4 for more information about the experiment setup and data used for the study.

The traditional statistical tool for establishing dependence structure between random variables in terms of their explicit (linear or non-linear) relationships is regression analysis, commonly carried out having estimated the model parameters by means of ordinary least squares regression (OLSR). However, in cases when the sample sizes are small, there are missing data and/or in the presence of strongly correlated variables, a development of the regression procedure is to base the analysis on Partial Least Squares (PLS). This initiative was taken by Wold [22] more than half a century ago and has proven an interesting idea [23,24,25,26,27,28] to come to terms with the numerous possible problems with regression based on OLS. There has been much development, [29,30,31,32,33,34] to mention a few, but also critical papers, e.g., [35,36,37]. There are also other approaches to cases with, e.g., imbalanced data. For example, for the detection of voice disorders in an imbalanced dataset, General Adversarial Network (GAN) and C-means clustering were used [38]. Nevertheless, the many problems with GANs are a well documented fact (see, e.g., [39,40]) mainly related to poor convergence of model parameters. Additionally, the cumbersome loss function implies that the model is very hard to train [41]. Thus, GAN is considered to be less appropriate for simulating activity data among people with dementia. Among Recurrent Neural Networks (RNNs) [42] are models and techniques for including time dependent data. By utilizing memory and so-called gates the Long-Short Term Memory Neural Network (LSTMNN) and the Gated Recurrent Unit (GRU) are methods which can learn how to filter out irrelevant data and remember the relevant. A problem here though is to optimally choose initial values for the weights of the oblivion gate and for the memory gate. Another disadvantage with these models is that the training needs very large amounts of data. PLS and its variants are to be considered as fast solutions [43] for parameter estimation, here, for simulation objectives. However, there are other methods of Machine Learning (ML) which can adapt to the data and produce simulations which more accurately correspond to the real data from a probabilistic point of view. Still, the price to pay is a higher computational complexity [44], more time-consuming simulation procedure, and sometimes convergence issues. For example, automatic pruning methods have been employed to reduce the complexity of AI models with multiple layers and a high number of parameters which are very difficult to deploy on resource-restricted platforms [45]. Similarly, the quantization approach has been proposed for shortening the number of trainable parameters and the associated computational effort of the entire implementation [46]. As previously discussed, the aforementioned methods are very useful for addressing overparametrized data structures. Nonetheless, in this case study, we only consider three features and their combinations which may not represent a high computational complexity. Indeed, PLSR is recognized by virtue of its computational efficiency [47] with applications in remote sensing environments [48] as exposed in this research. In ML, the complexity of methods is rarely below a polynomial time while in PLSR the complexity of methods is typically below the polynomial time. With PLSR the gain is a robust method with quick delivery. Below follows a very brief introduction of PLS, but a more thorough presentation of the field can be found in, e.g., [26,27,28].

The notion of partial least squares regression (PLSR) includes multiple regression, principal components analysis (PCA), and principal components regression (PCR) [28]. PCA allows pinpointing the smallest number of uncorrelated features, which reduces the complexity of the prediction model. Linear PLSR without intercept may be formally presented as follows. In Equation (1), the model is specified. For given *n* observations of covariates, *X*, (*m* variables) and *n* corresponding observations of responses, *Y*, (which can be *k* variables) the object is to minimize ∥ϵ∥ such that

(1) $$Y=TBQ^{T}+\epsilon$$

where $X=TP^{T}+\epsilon_{1}^{T}$, $Y=UQ^{T}+\epsilon_{2}^{T}$, *X* is an n×m matrix, *Y* is an n×k matrix, *T* is an n×a matrix, *P* is an m×a matrix, *U* is an n×a matrix, *Q* is an m×a matrix, *B* (regression coefficients), ϵ, $\epsilon_{1}$, $\epsilon_{2}$ (residuals) are n×1 vectors, ∥·∥ is the Euclidean norm and $\cdot^{T}$ denotes the matrix transpose. Determination of T,P,U and *Q* may be achieved by deploying the Nonlinear Iterative Partial Least Squares (NIPALS) method. This is an iterative procedure that converges to the optimal solutions for the least squares problem, which minimizes ∥ϵ∥ under the condition either of leaving out column vectors of *T* and *U* which do not correspond to variables with small eigenvalues or by systematically, for each iteration in the procedure, carry out an Analysis of Variance (ANOVA) based on resampling by means of a bootstrap method, which determines whether all coefficients in the model are significantly non-zero or not. Regarding the generalization ability, Hao and Chen [49] and Zheng et al. [50] proposed an interesting use of transferable feature learning and instance-level adaptation for visual recognition; in this case, the transferability of general features is achieved by calculating the regression coefficients *B* of each ADL while replacing the covariates *X* by the corresponding synthetic observations derived from the simulator when interacting with the user.

### Assessment of Model and Data

For ordinary least squares regression assumptions such as normality of residuals and independence between covariates should be checked. Regarding corresponding assumptions for PLSR one should check independence and equal distribution of the residuals. Independence is here examined with the Durbin–Watson test by rejecting for large values of the statistic in Expression (2)

(2) $$\frac{\sum_{i=2}^{n}{\left(\epsilon_{i}-\epsilon_{i-1}\right)}^{2}}{\sum_{i=1}^{n}\epsilon_{i}^{2}}$$

according to the $\chi^{2}$-distribution with n−1 degrees of freedom. The determination coefficient $R^{2}$ is calculated for learning about how much of the variation in the response that is explained by the model. Additionally, the Prediction Residuals Sum of Squares (PRESS) [51] as presented in Expression (3)

(3) $$diag\left({\left(Y-\hat{Y}\right)}^{T}\left(Y-\hat{Y}\right)\right)$$

is calculated to give some idea about the predictive quality of the model where *Y* is the observed response and $\hat{Y}$ is the predicted response according to the model. This evaluation measure is a common indicator of the optimal number of covariates. However, it is sometimes considered to be awkward due to the very large number of calculations required to be determined [52,53].

Apart from modelling, there is also a need for methodology to distinguish potential differences between real and synthetic data. To this end, a paired sample Wilcoxon test is performed for each pair of real and synthetic activity variables [54]. For the variables $X_{1}$ and $X_{2}$, the hypothesis as in Expression (4)

(4) $$\left\{\begin{array}{c} H_{0}:p=\frac{1}{2}\\ H_{1}:p\neq \frac{1}{2} \end{array}\right.$$

is to be tested where $p=P(X_{1,i}+X_{1,j}>X_{2,i}+X_{2,j})$, $X_{1,1},X_{1,2},\ldots ,X_{1,n}$ are observations of $X_{1}$ and $X_{2,1},X_{2,2},\ldots ,X_{2,n}$ are observations of $X_{2}$. Thus, the statistic

(5) $$T^{+}=\#\left\{X_{1,i}+X_{1,j}>X_{2,i}+X_{2,j}:i,j=1,\ldots ,nandi\leq j\right\}$$

is calculated as given in Equation (5). This is rejected for values $T^{+}=t$ such that $F_{T^{+}}\left(t\right)>1-\frac{\alpha }{2}$ or $F_{T^{+}}\left(t\right)<\frac{\alpha }{2}$ for a size α test. Here, $F_{T^{+}}$ denotes the approximate cumulative distribution function (cdf) [55]

(6) $$F_{T^{+}}\left(t\right)\approx \Phi \left(\omega \right)+\frac{\left(3n^{2}+3n-1\right)\omega \left(\omega^{2}-3\right)}{10n(n+1)(2n+1)}\cdot \frac{d\Phi (s)}{ds}{|}_{s=\omega }$$

of $T^{+}$ as given in Equation (6) where Equation (7) specifies the notation

(7) $$\omega =\frac{4t+2-n(n+1)}{\sqrt{\frac{8}{3}n\left(n+1\right)\left(2n+1\right)}}$$

and Φ(s) is the standard normal cdf. For further information about all these methods, please consider standard texts such as [56,57].

## 4. Experiment Definition

**Figure 1:**
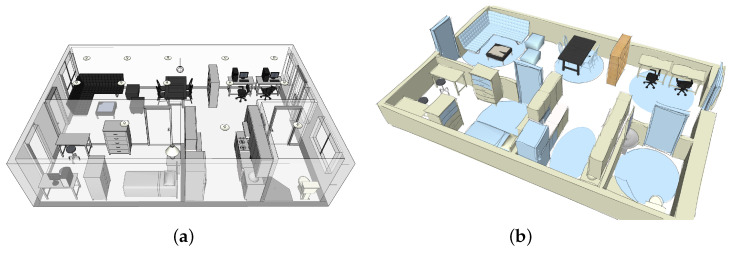
(**a**) The HINT layout. (**b**) The sensing capabilities of HINT.

The real environment is the Halmstad Intelligent Home (HINT) located in Halmstad, Sweden [58,59], which is supplied with contact/touch sensors recognizing occupancy in chairs and sofas. This apartment is also provided with Passive Infrared (PIR) sensors located in different spaces, thereby facilitating the continuous monitoring of motion and occupancy of subjects staying in their settings. Likewise, there are switches detecting the opening/closing of different home appliances and furniture doors installed in this home. The real architecture and floor plant layout of HINT are depicted in Figure 1. HINT is then suitable for underpinning two main purposes: (i) parsing out the ADLs performed by PwD over time and (ii) designing anticipated interventions for assisting PwD in case of severe health decline.

HINT was virtually modelled by IE Sim, a flexible interactive simulation software developed by Synnott et al. [60] considering the current sensor network placement within the home and the floor plan (Figure 1). The simulator also emulated the furniture items available in the real environment to make the representation more realistic and then collect activity duration data with better approximation to the real-world pattern.

**Figure 2:**
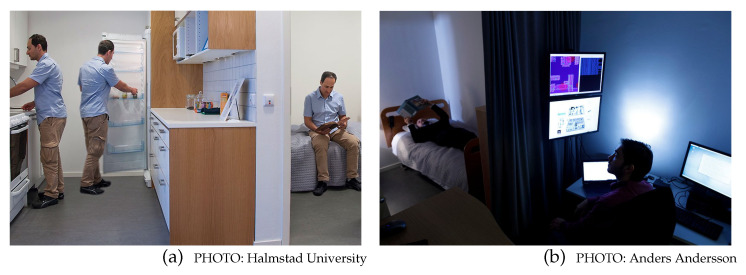
(**a**) A participant undertaking some of the ADLs in the kitchen and bedroom. (**b**) Human behaviour monitoring at HINT.

Whilst a huge amount of research has been carried out in the area of sensor-based human activity recognition, there is a lack of standardization in terms of methodology, particularly around the types of activities investigated and how performance of models is evaluated [61]. Currently, there is no recognised taxonomy of activities that should be investigated [18]. However, many previous works have focused on ADLs as described by Katz [62] to define a list of basic and necessary activities. Considering the lack of a standard taxonomy for activities, the ADLs selected within this work have been broadly mapped across these 6 activities including bathing, dressing, toileting, transferring, and feeding. As part of the experiment, eleven subjects were requested to undertake 8 ADLs (Stay in bed, Use restroom, Make breakfast, Get out of home, Get cold drink, Stay in the office, Get hot drink, and Cook dinner) while staying at HINT (Figure 2). Additionally, the same participants were asked to perform the ADLs using the IE Sim simulator. The following list of instructions was provided to guide the user on the sequence of tasks during the simulation:

*Previous general commands*

– Please carefully read the tasks to be carried out in each activity.
– Please feel free to re-read the instructions of each activity if necessary.
– Please press the Start/Stop switch when an activity is finished.
– Please lock the door upon crossing through.
– Please switch off each household appliance after use.

*ADLs description*

**ADL 1: Stay in bed**
  You can remain in bed as long as you wish. The maximum time is 2 min. After this, you have to get out of the bedroom, lock the door, and press the Start/Stop switch.
**ADL 2: Use restroom**
  You can use the hand-washing sink and/or toilet if you need. After this, please get out of the bathroom, lock the door, and press the Start/Stop switch.
**ADL 3: Make breakfast**
  You have to cook something for breakfast. Besides, you can select between milk and cereals or coffee. However, it is also possible to prepare both if you want. After this, move the bowl up on the dining table, sit down, and press the Start/Stop switch.
**ADL 4: Get out of home**
  You can decide to leave the house from the courtyard door or from the front door. When you are in outdoors, please push the Start/Stop switch.
**ADL 5: Get cold drink**
  You can take the drink from the refrigerator or serve plain water. After this, put the poured glass on the kitchen table and press the Start/Stop button.
**ADL 6: Stay in the office**
  Please proceed to the office and push the Start/Stop button.
**ADL 7: Get hot drink**
  You can select between preparing coffee or tea. After this, put the poured cup on the kitchen table and push the Start/Stop button.
**ADL 8: Cook dinner**
  Please make soup. Put the served bowl on the kitchen desk and push the Start/Stop button.

The results were two datasets (synthetic and real) containing a compilation of sensor events per ADL with each related to a time mark, the activated sensors (e.g., contact/touch, PIR, opening/closing), sensor identification code, and status (open or closed). Following this, three indicators were estimated: ADL duration, number of events per ADL, and number of events per sensor type per ADL. Previously, the datasets were cleaned by removing outliers deriving from errors during the execution of the experiments, thereby increasing the fit and predictive ability of PLSR models depicted in next section.

## 5. Results and Discussion

The ADLs performed by the subjects while staying at HINT are represented by more than 900 real sensor activations (μ=1.93; $\sigma^{2}=3.45$ min per subject). On the other hand, approximately 1100 synthetic sensor events were derived from interactions with the virtual IE Sim environment (μ=1.675; $\sigma^{2}=13.80$ min per participant). Overall, 36 synthetic samples (10 activity durations, 10 events per activity, and 16 events per sensor per activity) were generated per each ADL. This represents a total of 288 samples in the whole experiment. Prior to undertaking the ADLs in IE Sim, the subjects first learned how to handle the software to diminish bias and flatten the learning curve. In this case, all the participants completed the ADLs satisfactorily in both real and synthetic homes. In fact, the average time spent by each subject carrying out all the ADLs was 8.69 ($\sigma^{2}=4.21$ min) and 13.93 ($\sigma^{2}=12.65$ min) at HINT and IE Sim, respectively. When comparing these metrics, it is evident that, in general, the time used by participants to complete all the ADLs in the simulator is significantly higher than the one spent in the real apartment (*T*-value = 4.24; *p*-value = 0.002; 95% CI for the difference: [2.49–7.99] min). The following sub-section will elucidate if this difference is also detected in all the ADLs individually. PLSR models will be applied to better approximate the simulated data to real activity durations in those ADLs with significant differences.

### 5.1. Contrasting Real and Synthetic Activity Duration

**Figure 3:**
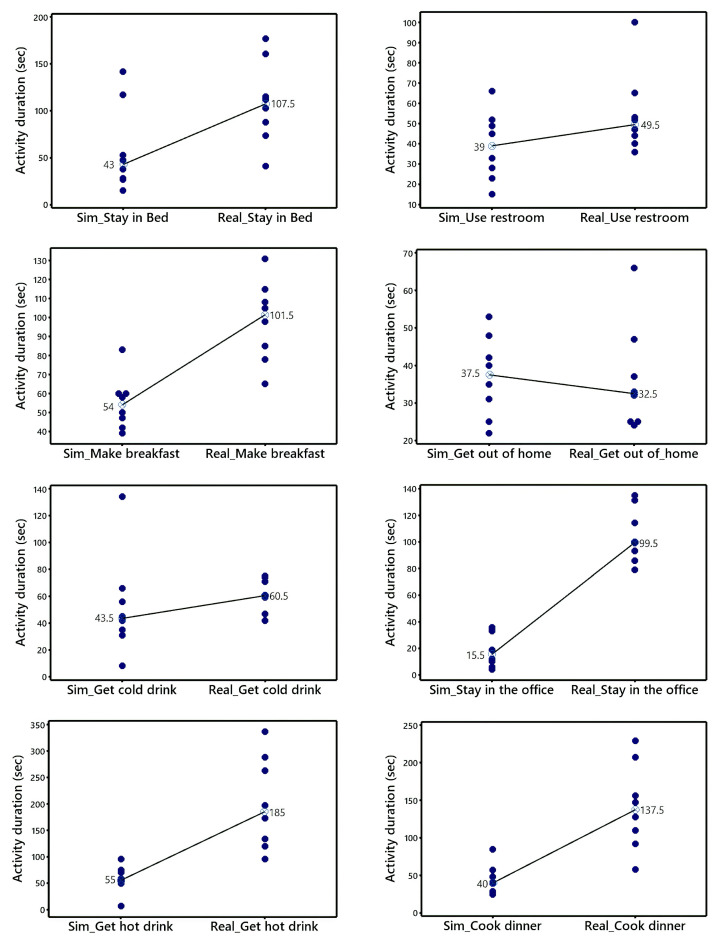
Differences between synthetic and real activity duration. The ADLs in the first row from the left are: *Stay in bed* and *Use restroom*; second row: *Make breakfast* and *Get out of home*; third row: *Get cold drink* and *Stay in the office*; and fourth row: *Get hot drink* and *Cook dinner*.

A two-sample Wilcoxon test (α=0.05) was applied to analyse the equivalence between real and synthetic activity duration in each of the eight ADLs defined in the experiment description. Thereby, it will be possible to detect those ADLs in which a statistical transformation is needed to better approximate the synthetic activity duration to the one in the real environment. Table 1 enlists the results of the comparison in terms of *p*-values, 95% confidence interval (CI) for the median difference, and the conclusions. The results are also graphed through individual value plots comparing the data distribution and medians between the synthetic and real observations (Figure 3).

The Wilcoxon test outcomes revealed significant differences (*p* value ≤ 0.05) between the simulated and real-world data for the activity duration indicator in five ADLs: *Use restroom*, *Make breakfast*, *Stay in the office*, *Get hot drink*, and, *Cook dinner*. It is then concluded that the simulator does not accurately represent the real intrinsic properties of users when performing these activities. This is also confirmed by the 95% confidence intervals (CI), which do not include zero, but show lean to the left. Mostly, the gaps have been detected in activities with a larger number of sensor events than those performed in the kitchen.

Looking into the results, it is evident that simulated activity duration data tend to be meaningfully minor compared to those from the real smart home. In this regard, it is important to identify the sensors triggering the gap as well as analysing the sequence of events within each ADL. Likewise, intrinsic factors of users may influence the ADL duration. For example, some subjects may need more training than others before performing the simulations in a natural way. Additionally, the participants may prepare meals in multiple manners and ADL durations could therefore evidence high variability. Likewise, external aspects (i.e., the time of the day) may also contribute to this difference. In fact, some simulators have been reported as incapable of capturing the natural variations of activity durations in the presence of these elements [60]. Another factor potentially influencing these outcomes is the dissimilarities between home layouts of real and simulated environments. The distances travelled by users through the avatar are not equivalent to those covered at the apartment, which may increase the deviations between the duration metrics.

On a different note, detailed and high-quality data is required for effectively training AR models so that they can identify behavioural patterns in the daily routine of PwD. Given the limitations of real data collection widely depicted in the introduction (see Section 1), training datasets are expected to be formed by synthetic observations mimicking the intrinsic properties of real data. In this regard, it is not advised to use data evidencing significant deviations from the real world; therefore, these results reveal the need for approximating these observations to those from the real smart homes to increase the prediction accuracy and other performance indicators of AR models. The data analysts are then recommended to use the PLSR equations to transform the data and then include them in the training datasets. If this is not carried out, the AR model will evidence poor performance in identifying significant changes in the duration of each ADL.

These results are comparable with those presented in Lee et al. [15]. Specifically, the authors used a stochastic analysis to evaluate the similarity of the synthetic datasets produced by Persim (3Da, 3Du, and 1.5) in contrast with those gathered from the real environment. The analysis was focused on the occurrence probability and the number of sensor events. In summary, the highest average similarity was found to be 81% (Persim 1.5). Similarly, Dahmen and Cook [7] used significance comparative analysis, Euclidean distance, and Dynamic Time Warping (DWT) to examine the realism of data derived from the SynSyn simulator. However, both assessments were made in general and not targeted to each ADL as our paper does. In fact, this study reveals great equivalence in *Stay in bed*, *Get out of home*, and *Get cold drink*, but significant disparities in the remaining ADLs, which may be hidden in a general analysis. Conversely, Alharbi et al. [14] employed the GAN-test method to verify how well the simulated samples mimic the real data distributions. In this case, F1 scores revealed perfect equivalence in all datasets and classes considering *run*, *still*, *walk*, *bike*, and *bus* activities. Nonetheless, our case study takes into account a larger number of ADLs that are also more complex to analyse. ADLs like *cook dinner*, *make breakfast*, and *get hot drink* are performed in several ways, which is proven by a variable number of sensor events and a plurality of event sequences. Additionally, Kamara-Esteban et al. [16] appraised the coherence of synthetic datasets with real measurements in both single-user and multiple-user scenarios considering the frequency and activity duration of five ADLs (Preparing breakfast, Washing dishes, Shower, Preparing dinner, and Do laundry). The authors deployed probability distributions and confidence intervals to evaluate the similarity. Nevertheless, a summary *p*-value statistic was not provided per each ADL so that disparities can be further categorized as significant or non-significant, thereby supporting the use of models approximating the synthetic observations to a more realistic behaviour as this study does. Other important related efforts described in Alshammari et al. [17], Park et al. [63], Synnott et al. [64], Ariani et al. [65], and McGlinn et al. [66] did not present any comparative analysis between synthetic and real data from ADLs in smart homes.

### 5.2. Transforming Synthetic Data to Predict Real Activity Duration

Simulated data are expected to emulate the real-world behaviour of PwD and then be useful to train AR models capable of detecting deviations or change points explaining the disease progress. Nonetheless, it is evident from Section 5.1 that some ADLs (*Use restroom*, *Make breakfast*, *Stay in the office*, *Get hot drink*, and *Cook dinner*) are significantly different in terms of activity duration when performed in both real and synthetic smart environments. In similar contexts, while Kamara-Esteban et al. [16], Alharbi et al. [14], and Lee et al. [15] did not use any method to transform the synthetic data before including them in the training datasets, Dahmen and Cook [7] proposed the use of 12 state hidden Markov models and probabilistic modelling techniques to transform the data; however, this study did not evidence how the models may vary depending on the ADL nature. Being aware of this, this Subsection presents PLSR models transforming the synthetic data to better approximate the real-world activity durations corresponding to these ADLs. The usability of each model was further assessed considering the assumptions explained in Section 3. Valid models were then described in terms of predictive power and fit to then demonstrate their capability for generating intricate real data that effectively complement the small-size datasets (built from the subjects staying at HINT). All the models were run on a PC with a 2.90 GHz Intel(R) Core(TM) i5-9400 CPU processor.

#### 5.2.1. Use Restroom

When modelling real activity duration, it is essential to identify synthetic factors highly contributing to the response variable. Table 2 depicts the ANOVA test deployed to this aim. It can be noted that Synthetic Activity Duration (${SAD}_{UR}$) (*p*-value = 0.021), Number of Events per Pressure Sensor (${NEPS}_{P}$) (*p*-value = 0.040), and the two-order interaction (${SAD}_{UR}*{NEPS}_{P}$) (*p*-value = 0.003) were concluded to be statistically significant on *Y* and can therefore be considered in the PLSR model. In fact, the logarithmic polynomial model (Equation (8)) was concluded to be appropriate for analysing the variability of activity duration when subjects use the restroom (*p*-value = 0.000; α=0.05).

On the other hand, the model presented in Equation (8) has also been evaluated from the goodness-of-fit and predictive ability perspectives (Table 3). In this respect, an important metric to study is the standard error of the estimate (S) which, in this instance, indicates that the average distance of the original observations from the model zone is about 0.639 s. This result is near 0, thereby demonstrating a close match for the prediction interval. Meanwhile, Table 3 postulates an $AdjR^{2}=97.40\%$ revealing a high explanatory power of the model proposed for *Use restroom*. Additionally, satisfactory predictive performance was proved through the PRESS statistic (4.51), which favours the use of this model in providing valid predictions that can complement the training datasets employed by the AR algorithms. Random noise was also discarded, as there is no meaningful difference between the $AdjR^{2}$ and $R^{2}\left(pred\right)$ (0.99%). From the scalability point of view, it is good to highlight that the computational run time for this model ranged from 0.630 to 0.690 s.

(8) $$\ln Y=0.1132*{SAD}_{UR}+0.298*{NEPS}_{P}-0.0077*{SAD}_{UR}*{NEPS}_{P}$$

Finally, the DW statistic was calculated to verify the interdependence assumption of residuals. As DW>DU (2.549 > 1.777), neither negative nor positive auto-correlation is evidenced in the group of random errors. In the meantime, homogeneity of variances was confirmed, thereby validating the adequacy of the PLSR model shown in Equation (8).

#### 5.2.2. Make Breakfast

Table 4 presents the results of the ANOVA test performed to detect synthetic variables that can support the prediction of time spent by the subjects when making breakfast. It is good to highlight that ${NEPA}_{MB}$ (*p*-value = 0.004) was identified as a good predictor because the number of tasks may vary from one person to the other as there are different ways of cooking; even without considering the ordering of events, which is intrinsic in each subject. Likewise, the significance of ${SAD}_{MB}$ (*p*-value = 0.004), also reported in Dahmen and Cook [7], explains how the specific characteristics of PwD can be captured through a simulator (e.g., IESim) to then be transformed for representing the real behaviour. Interestingly, when coupling these terms ${SAD}_{MB}*{NEPA}_{MB}$ (*p*-value = 0.014), it was further noticed how each ADL (in this case, make breakfast) is differently performed by each individual, thereby underpinning the finding postulated by Fortino et al. [67] regarding the wide range of PwD profiles that may be encountered in the real world. Learning about these ADLs appropriately is of utmost importance for modelling activity durations more accurately. In this instance, the final outcome is a quadratic polynomial expression (Equation (9)) capable of predicting the time invested by the inhabitants of the HINT smart home when preparing breakfast (*p*-value = 0.000).

The performance indicators illustrated in Table 5 further detail the characteristics of the model (Equation (9)) with regards to the ability to forecast the real activity duration of inhabitants when preparing breakfast based on the simulator outputs. The findings point out a very small divergence between the $AdjR^{2}$ and $R^{2}\left(pred\right)$ (1.07%), therefore discarding the presence of overfitting effects hindering the applicability of the model to complement the datasets used for training the associated AR algorithms. Notably, an excellent fit is provided by the attained quadratic expression as $AdjR^{2}$ is over 90%. No less important are the low values of *S* (1.206) and PRESS (20.04), which are accounted by the overlapping of fitted data with the prediction interval.

(9) $$\sqrt{Y}=0.2567*{SAD}_{MB}+0.930*{NEPA}_{MB}-0.0249*{SAD}_{MB}*{NEPA}_{MB}$$

Every PLSR model needs to be appraised in search of potential abnormalities or random errors that may invalidate the predictions made by the model in presence of new synthetic entries. Consequently, the randomness of residuals was scanned via the DW indicator at a 5% significance level. As the DW>DU (2.064 > 1.777), sufficient evidence has been collected to reject potential inter-dependencies among the residuals. Complementary to this result, no proof of unequal variances was detected in the sample which, in addition to the foregoing, confirms the practicality of the model in real applications including the one related to the AR models for *Prepare breakfast*. In this case, the computational time invested in this model fluctuated between 0.480 and 0.550 s.

#### 5.2.3. Stay in the Office

Another challenging ADL to model based on synthetic data is *Stay in the office*. The time invested by individuals staying at the smart homes may differ from one to the other based on a plethora of extrinsic and intrinsic factors. Despite this, as seen in other ADLs, the number of events per activity (${NEPA}_{SIO}$; *p*-value = 0.000) and synthetic activity duration (${SAD}_{SIO}$; *p*-value = 0.006) were held to be good regressors of the time invested by the individuals when staying in the office (Table 6). In fact, as also evidenced in other ADLs, the interplay between these simulated variables was also concluded as significant on the response variable *Y* and can be included in the predictive model (Equation (10)).

The proposed equation (Equation (10)) satisfies all the conditions for further use in real AR applications. First, both $AdjR^{2}$ and $R^{2}\left(pred\right)$ are over 90%, thus indicating that this quadratic polynomial expression explains a great proportion of the activity duration variation in *Stay in the office* and in the meantime is excellent for making predictions of the response variable. This allows us to create an approximation of real data without a time-consuming recruitment process and substantial investments. Training AR classifiers required datasets with enough size so that the algorithms can effectively learn about the human behaviour in a particular ADL [68]. Nonetheless, it is advised to collect a pre-sample of real data (approximately 10 individuals) to estimate the effects of new office layouts and other critical factors on the activity duration. In the near future, other synthetic features may be explored to increase the fitting and prediction power of the model. On a different tack, the small distance between $AdjR^{2}$ and $R^{2}\left(pred\right)$ (3.51%) proves that all of the terms do not substantially increase the model bias, which makes it useful for complementing the small-sized datasets constructed from the smart home environments. In addition to this body of findings, it is good to highlight the small values of *S* (2.431) and PRESS (64.83) that provide good support for the high prediction performance statement (Table 7). An important aspect is the run time, which varied from 0.580 to 0.610 s.

(10) $$\sqrt{Y}=2.227*{NEPA}_{SIO}-0.040*{SAD}_{SIO}*{NEPA}_{SIO}$$

Ultimately, the presence of auto-correlation in the adjacent residuals of the PLSR model proposed for describing the duration of *Stay in the office* was evaluated via the Durbin–Watson test at a 5% significance level. This discards the fact that the PLSR equation can miscalculate the standard error of the coefficients and therefore show them significantly when they are really not. As D>DU (1.814 > 1.777), no correlation exists among the random errors. Furthermore, no heterogeneity was observed in the residuals, which favours the adequacy of the model when used for underpinning the training of the correspondent AR classifier.

#### 5.2.4. Get Hot Drink

Table 8 presents the significance analysis of potential predictors included in Equation (11). In this instance, a *p*-value of 0.000 proves that the PLSR model proposed in Equation (11) is suitable for describing the variations of real activity duration *Y* when getting a hot drink (*p*-value = 0.000; α=0.05). This is supported features significantly contributing to the prediction of *Y*: Synthetic Activity Duration (${SAD}_{GHD}$) (*p*-value = 0.004), the Number of Events per Activity (${NEPA}_{GHD}$) (*p*-value = 0.004), and the interaction between these variables (${SAD}_{GHD}*{NEPA}_{GHD}$) (*p*-value = 0.002). All these synthetic factors were considered in a polynomial quadratic PLSR formula modelling the time spent by a person getting hot drinks at a real home environment.

The model attained in Equation (11) evidences very good performance in both fit and predictive power domains (Table 9). First, the standard deviation *S* was estimated to be very small (2.645), and it can therefore be deemed as a symptom of model adequacy regarding the description of activity durations *Y* in *Get hot drink*. Moreover, Table 9 shows an $AdjR^{2}=96.51\%$, which demonstrates an excellent fit of Equation (11) with respect to the original observations. On a different tack, adequate predictive power was demonstrated as the PRESS indicator (77.27) was found to be very low. In a similar vein, an insignificant difference (1.32%) was noted between the $AdjR^{2}$ and $R^{2}\left(pred\right)$, thereby rejecting the overfitting problem in the model. From the applicability perspective, it is good to stress the low computational time invested by the data analysts to derive the model (0.580 to 0.690 s).

(11) $$\sqrt{Y}=0.2811*{SAD}_{GHD}+2.191*{NEPA}_{GHD}-0.0386*{SAD}_{GHD}*{NEPA}_{GHD}$$

The usability of the PLSR model proposed in Equation (11) is validated through the randomness and equal variance assumption (Equation (3)). The interdependence hypothesis was evaluated by employing the Durbin–Watson test (Equation (2)). In this case, the observed DW (2.981) was concluded to be higher than the DU limit (1.777), and it is thus not evidenced by the presence of significant auto-correlations among the random errors of the model. In the meantime, the homogeneity assumption was assessed; however, no sufficient support was detected against the null hypothesis. The above-mentioned reasons then provide a statistical base underpinning the model deployment in the real world.

#### 5.2.5. Cook Dinner

The ANOVA test (Table 10) indicates that the PLSR model proposed in Equation (12) is adequate to explain the variability of real activity duration *Y* when cooking dinner (*p*-value = 0.000; α=0.05). More specifically, the Synthetic Activity Duration (${SAD}_{CD}$) (*p*-value = 0.002), the Number of Events per Chair Pressure Sensor (${NEPS}_{CHP}$) (*p*-value = 0.001), and the interaction between these factors (${SAD}_{CD}*{NEPS}_{CHP}$) (*p*-value = 0.007) were concluded to be good predictors of *Y* and can therefore be employed for modelling this response variable. In this instance, a logarithmic PLSR expression was found to be the most effective alternative for describing the time period taken by each subject when preparing dinner. It took from 0.510 to 0.690 s to generate the PLSR model.

Upon assessing the predictive ability and fit inherent to the model in Equation (12), it is worth noting that the standard deviation of the data points around the fitted observations *S* is close to 0 (0.734 s), which demonstrates that there are no significant distances between the original *Y* values and the fitted data. We can accordingly postulate that the model (Equation (12)) is appropriate to describe the activity duration *Y* patterns corresponding to *Cook dinner*. Additionally, as depicted in Table 11, a high proportion of the variation in *Y* ($AdjR^{2}=97.74\%$) is described by Equation (12), thereby providing good support for the goodness-of-fit hypothesis. On a different note, the PRESS statistic (13.55) was calculated to be small, thereby underpinning a satisfactory predictive ability of the model. Similarly, the predictive $R^{2}\left(pred\right)$ (93.11%) proves that the polynomial model predicts the response for new observations at high performance and can be hence categorized with excellent predictive ability. No less important is the small gap between the $AdjR^{2}$ and $R^{2}\left(pred\right)$ (4.63%), which discards overfitting problems and confirms that the model is effective for estimating new real durations of *Cook dinner* activity when entering new ${SAD}_{CD}$ and ${NEPS}_{CHP}$ values into Equation (12).

(12) $$\ln Y=0.1888*{SAD}_{CD}+1.255*{NEPS}_{CHP}-0.0311*{SAD}_{CD}*{NEPS}_{CHP}$$

Lately, the independence assumption was verified through the Durbin–Watson (DW) statistic (Equation (2)). As the number of regressors denoted by $k^{\prime }$ = 2 (${SAD}_{CD}$ and ${NEPS}_{CHP}$) and the sample size *n* = 8, the respective theoretical printed bounds (without intercept) are dL = 0.371 and dU = 1.777. Comparing the observed DW value (1.888) with the parameters dL and dU at the 5% level of significance, it is proven that no positive/negative auto-correlation exists among the model residuals (DW>dU). Likewise, no heterogeneity of residuals was detected in this case, thereby validating the suitability of the proposed model.

## 6. Conclusions

Monitoring the daily living of PwD along the time supports the early diagnosis and treatment by healthcare systems, which contributes to increasing the life quality of these patients while alleviating the economic burden caused by this disease. The cornerstone is the effective automatic recognition of ADLs whose intrinsic properties and evolution provide a comprehensive overview of how dementia has progressed over time. Nonetheless, the performance of these recognition models is greatly based on the availability of intricate and suitable data. Adversely, there is accompanying difficulty in achieving formal ethical approval for gathering massive amounts of data while recruitment processes are costly, resource-consuming, and characterized by low engagement rates.

Simulation tools have arisen as a more efficient solution to obtaining synthetic data complementing the real datasets. The principal barrier hindering the potential applicability of these simulators is that synthetic data for critical behavioural variables (e.g., activity duration) have been concluded to be substantially divergent from the real observations in ADLs with a large number of tasks. This outcome is underpinned by differences in user profiles, home layouts, and background in the use of simulators. In this respect, significant dissimilarities were detected between the real and simulated activity durations of *Use restroom*, *Make breakfast*, *Stay in the office*, *Get hot drink*, and *Cook dinner*. In this case, the “activity duration” variable has been targeted considering its capability of evidencing behavioural changes in PwD, which may be an indicator of health decline as a result of a neurological deterioration process.

In this paper, we have proposed the use of PLSR models to transform the synthetic variables derived from the IE Sim simulator into a better approximation of real activity durations calculated from stays at the HINT smart home. It has aimed to bridge the previous gaps regarding research contrasting real and synthetic data. It goes beyond other reported approaches such as the SynSys algorithm [7], which does not consider the particular nature of each ADL, an aspect addressed by the proposed PLSR models.

Two primary contributions stem from this research. The main outcome has been the creation of predictive models providing a more accurate transformation of simulated data for describing real activity durations. In particular, logarithmic and quadratic polynomial models were defined for predicting the real duration of the aforementioned ADLs. All the models were found to provide good fitting ($AdjR^{2}>90\%$) and predictive power ($R^{2}\left(pred\right)>90\%$; MaxS<3; MaxPRESS<100) whilst satisfying the PLSR adequacy assumptions. Therefore, they can be used for complementing the small-sized datasets employed for training the AR algorithms. A secondary contribution of this work has been the identification of the main synthetic predictors for real activity duration in each ADL, which helps to elucidate the intrinsic properties of each activity and how this metric may vary from one user to the other. Specifically, the Synthetic Activity Duration, Number of Events per Activity, Number of Events per Sensor per Activity, and their interactions were concluded to be the most popular regressors of real activity durations since they capture the intrinsic properties of human behaviour when staying at home. Of course, the results presented here may be limited to the specific layouts of IESim and HINT, as well as the experience of the inhabitants in the use of the simulator. Hence, it is recommended to first train the users on how to perform the ADLs virtually until overcoming the learning curve.

Future works will consider the sequence of tasks (sensor activations) performed by the subjects within each ADL. Thereby, it will be possible to discriminate and model the intrinsic properties of human behaviour. In a similar vein, it is important to explore how the sensor event ordering can be included in the PLSR models and whether the predictive ability and fit can be upgraded for producing a better approximation of the real activity duration. Another pathway to investigative is the application of other prediction algorithms, including Random Forest and Naïve Bayes, to perform comparative analysis with a focus on the ADLs with significant differences between real and synthetic activity durations as identified in this work.

## Figures and Tables

**Table 1:** Two-sample Wilcoxon test results.

ADL	*p*-Value	95% CI for the Difference (sec)	Conclusion
Stay in bed	0.093	[−5; 100]	Statistically similar
Use restroom	0.050	[−34; −1]	Statistically different
Make breakfast	0.012	[−66; −22]	Statistically different
Get out of home	0.889	[−12; 16]	Statistically similar
Get cold drink	0.161	[−36; 7]	Statistically similar
Stay in the office	0.012	[−104; 64]	Statistically different
Get hot drink	0.018	[−236; −62]	Statistically different
Cook dinner	0.012	[−159; −44]	Statistically different

**Table 2:** ANOVA results for the *Use restroom* PLSR model.

Source	DF	Contribution	Adj SS	Adj MS	*F*-Value	*p*-Value
Regression	3	98.32%	123.55	41.18	100.79	0.000
${SAD}_{UR}$	1	3.61%	4.54	4.54	11.11	0.021
${NEPS}_{P}$	1	2.46%	3.09	3.09	7.50	0.040
${SAD}_{U}*{NEPS}_{P}$	1	9.47%	11.90	11.90	29.12	0.003
Error	5	1.62%	2.043	0.4087		
Total	8	100%	125.60			

**Table 3:** Predictive ability and fit of *Use restroom* PLSR model.

S	$R^{2}$	Adj $R^{2}$	PRESS	$R^{2}$ (Pred)
0.639	98.37%	97.40%	4.51	96.41%

**Table 4:** ANOVA results for the *Make breakfast* PLSR model.

Source	DF	SS	Contribution	Adj SS	Adj MS	*F*-Value	*p*-Value
Regression	3	777.21	99.07%	777.72	259.24	178.08	0.000
${SAD}_{MB}$	1	739.72	94.23%	37.46	37.46	25.73	0.004
${NEPA}_{MB}$	1	18.33	2.33%	37.96	37.96	26.08	0.004
${SAD}_{MB}*{NEPA}_{MB}$	1	19.66	2.50%	19.66	19.66	13.51	0.014
Error	5	7.27	0.93%	7.27	1.45		
Total	8	785.00	100%				

**Table 5:** Predictive ability and fit of *Make breakfast* PLSR model.

S	$R^{2}$	Adj $R^{2}$	PRESS	$R^{2}$ (Pred)
1.206	99.07%	98.52%	20.04	97.45%

**Table 6:** ANOVA results for the *Stay in the office* PLSR model.

Source	DF	SS	Contribution	Adj SS	Adj MS	*F*-Value	*p*-Value
Regression	2	801.53	95.76%	801.53	400.76	67.80	0.000
${NEPA}_{SIO}$	1	702.81	83.97%	333.92	333.91	56.49	0.000
${SAD}_{SIO}*{NEPA}_{SIO}$	1	98.72	11.79%	98.72	98.72	16.70	0.006
Error	6	35.47	4.24%	35.47	35.47	1.45	1.45
Total	8	837.00	100%				

**Table 7:** Predictive ability and fit of *Stay in the office* PLSR model.

S	$R^{2}$	Adj $R^{2}$	PRESS	$R^{2}$ (Pred)
2.431	95.76%	94.35%	64.83	92.25%

**Table 8:** ANOVA results for the *Get hot drink* PLSR model.

Source	DF	SS	Contribution	Adj SS	Adj MS	*F*-Value	*p*-Value
Regression	3	1570.99	97.82%	1570.99	523.66	74.80	0.000
${SAD}_{GHD}$	1	1287.84	80.19%	184.11	184.11	26.30	0.004
${NEPA}_{GHD}$	1	13.23	0.82%	174.71	174.71	24.95	0.004
${SAD}_{GHD}*{NEPA}_{GHD}$	1	269.92	16.81%	269.92	269.92	38.55	0.002
Error	5	35.01	2.18%	35.01	7.001		
Total	8	1606.00	100%				

**Table 9:** Predictive ability and fit of *Get hot drink* PLSR model.

S	$R^{2}$	Adj $R^{2}$	PRESS	$R^{2}$ (Pred)
2.645	97.82%	96.51%	77.27	95.19%

**Table 10:** ANOVA results for the *Cook dinner* PLSR model.

Source	DF	SS	Contribution	Adj SS	Adj MS	*F*-Value	*p*-Value
Regression	3	188.24	98.59%	188.24	62.74	116.28	0.000
${SAD}_{CD}$	1	163.16	85.45%	19.02	19.02	35.25	0.002
${NEPS}_{CHP}$	1	14.502	7.60%	24.28	24.28	45.00	0.001
${SAD}_{CD}*{NEPS}_{CHP}$	1	10.57	5.54%	10.57	10.57	19.59	0.007
Error	5	2.698	1.41%	2.69	0.53		
Total	8	190.93	100%				

**Table 11:** Predictive ability and fit of *Cook dinner* PLSR model.

S	$R^{2}$	Adj $R^{2}$	PRESS	$R^{2}$ (Pred)
0.734	98.59%	97.74%	13.15	93.11%

## Data Availability

Not applicable.

## Abbreviations

The following abbreviations are used in this manuscript:

ADLs	Activities of Daily Living
AI	Artificial Intelligence
ANOVA	Analysis of Variance
AR	Activity Recognition
CNN	Convolutional Neural Network
DF	Degrees of Freedom
DTW	Dynamic Time Warping
DW	Durbin Watson
GANs	Generative Adversarial Networks
HINT	Halmstad Intelligent Home
HMM	Hidden Markov Model
ML	Machine Learning
MS	Mean Square
NEA	Number of Events per Activity
${NEPA}_{GHD}$	Number of Events per Activity for Get Hot Drink
${NEPA}_{MB}$	Number of Events per Activity for Make Breakfast
${NEPA}_{SIO}$	Number of Events per Activity for Stay in the Office
${NEPS}_{P}$	Number of Events per Pressure Sensor
${NEPS}_{CHP}$	Number of Events per Sensor (Chair Pressure)
NESA	Number of Events per Sensor per Activity
NIPALS	Nonlinear Iterative Partial Least Squares
OLSR	Ordinary Least Squares Regression (OLSR)
PCA	Principal Components Analysis
PCR	Principal Components Regression
PIR	Passive Infrared
PLS	Partial Least Squares
PLSR	Partial Least Squares Regression
PRESS	Prediction Residuals Sum of Squares
PwD	People with Dementia
SAD	Smoking Activity Dataset
${SAD}_{CD}$	Synthetic Activity Duration for Cook Dinner
${SAD}_{GHD}$	Synthetic Activity Duration for Get Hot Drink
${SAD}_{MB}$	Synthetic Activity Duration for Make Breakfast
${SAD}_{SIO}$	Synthetic Activity Duration for Stay in the Office
${SAD}_{UR}$	Synthetic Activity Duration for Use Restroom
SHL	Sussex-Huawei Locomotion
SS	Squared Sum
UTI	Urinary Tract Infection
WHO	World Health Organization
